# Molecular Imaging in Gynecology: Beyond Cancer

**DOI:** 10.2967/jnumed.124.267546

**Published:** 2024-07

**Authors:** Joni Sebastiano, Cindy Rodriguez, Zachary V. Samuels, Kristen Pepin, Brian M. Zeglis

**Affiliations:** 1 Department of Chemistry, Hunter College, City University of New York, New York, New York;; 2 Department of Radiology, Memorial Sloan Kettering Cancer Center, New York, New York;; 3 Ph.D. Program in Biochemistry, Graduate Center of City University of New York, New York, New York;; 4 Ph.D. Program in Chemistry, Graduate Center of City University of New York, New York, New York;; 5 Department of Obstetrics and Gynecology, Weill Cornell Medicine, New York, New York; and; 6 Department of Radiology, Weill Cornell Medical College, New York, New York

**Keywords:** molecular imaging, PET, SPECT, fluorescence imaging, intraoperative imaging, MRI

## Abstract

Gynecological pathologies account for approximately 4.5% of the overall global disease burden. Although cancers of the female reproductive system have understandably been the focus of a great deal of research, benign gynecological conditions—such as endometriosis, polycystic ovary syndrome, and uterine fibroids—have remained stubbornly understudied despite their astonishing ubiquity and grave morbidity. This historical inattention has frequently become manifested in flawed diagnostic and treatment paradigms. Molecular imaging could be instrumental in improving patient care on both fronts. In this Focus on Molecular Imaging review, we will examine recent advances in the use of PET, SPECT, MRI, and fluorescence imaging for the diagnosis and management of benign gynecological conditions, with particular emphasis on recent clinical reports, areas of need, and opportunities for growth.

Gynecological diseases represent a tremendous global health burden (1). Within this umbrella, cancers have rightly received a great deal of focus, but nonmalignant disorders—such as endometriosis, polycystic ovary syndrome (PCOS), and uterine fibroids—have long been understudied and underaddressed. Although societal factors (i.e., the historically patriarchal nature of science and medicine) certainly play a factor in this neglect, the very low mortality rates associated with these conditions are almost surely responsible as well. Yet what these disorders lack in mortality, they more than make up for in ubiquity and morbidity. For example, it is estimated that up to 21% of women worldwide have PCOS, and endometriosis affects roughly 10% of women of reproductive age globally (2*,*^3^). Furthermore, these disorders exact a considerable toll on women, with symptoms ranging from menstrual cycle irregularity and gastrointestinal distress to chronic pain and infertility (4).

Current strategies for the diagnosis and treatment of benign gynecological disorders are lacking. The clinical gold standard for the detection of endometriosis, for example, is laparoscopic surgery, an invasive procedure whose inherent limitations—that is, expense, pain, and operator bias—contribute to a 5- to 7-y diagnostic delay for the condition (5). With respect to treatment, surgical resection remains a common strategy for uterine fibroids and adenomyosis, though less invasive options exist as well (6). Molecular imaging has the potential to be instrumental in improving care on both fronts. Noninvasive imaging agents could offer diagnostic options that are safer, cheaper, and more effective. Similarly, theranostic imaging could help monitor the efficacy of emergent molecular therapies (e.g., the use of progesterone receptor modulators for uterine fibroids), whereas intraoperative imaging could help improve accuracy during the resection of benign lesions such as uterine fibroids and endometriomas (7).

Recent years have played witness to increasing interest in the use of molecular imaging in patients with benign gynecological disorders. However, the literature is somewhat fragmented, and the intersection of these 2 fields has not yet been collectively reviewed. In this installment of the Focus on Molecular Imaging series, we will examine recent advances in the use of PET, SPECT, MRI, and fluorescence imaging for the diagnosis and management of these conditions (Fig. 1). Although both preclinical and clinical results will be covered, particular emphasis will be placed on recent human trials as well as areas of need and opportunities for growth. It is our sincere hope that this work will not only highlight exciting extant results but also inspire new work by bringing attention to the gaps that can be filled by repurposing old tools or developing new ones.

**FIGURE 1.**
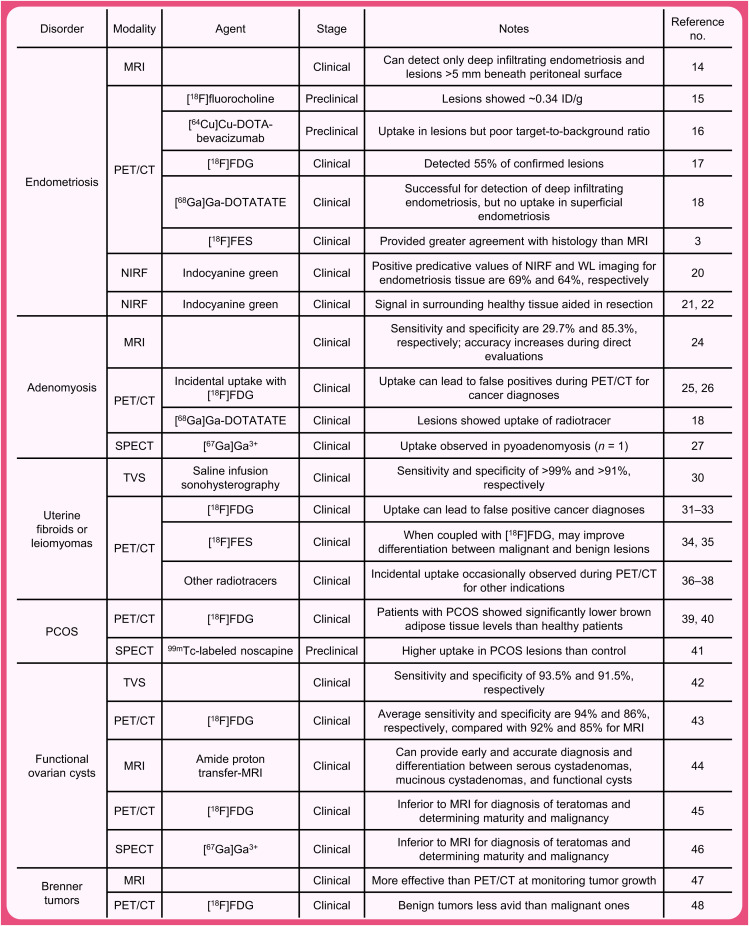
Studies cited for diagnosis and management of conditions.

Before we begin, it is important to recognize that one imaging modality—ultrasonography—has long been commonplace in gynecology. Ultrasonography is easily the most often-used noninvasive diagnostic tool in the field, helping clinicians visualize both normal and aberrant pelvic anatomy. Yet despite its ubiquity, ultrasound is not without problems, as it suffers from suboptimal specificity for some conditions (e.g., endometriosis and adenomyosis) and is susceptible to operator, technique, and interpretation biases (8). We have chosen not to cover ultrasound imaging in this review, primarily because its outsized presence in the field threatens to swamp our discussion of other emergent modalities but also because, strictly speaking, it is more of an anatomic than molecular imaging methodology. However, excellent reviews on its present and future role in the management of benign gynecological conditions can be found elsewhere (8).

## ENDOMETRIOSIS

Endometriosis is a chronic female reproductive disorder characterized by the growth of endometrial tissue outside the uterus (9). Most endometriosis lesions grow on the outer perimetrium, fallopian tubes, and ovaries. However, in some cases—known as deep infiltrating endometriosis—endometrial tissue may grow on the bowels, rectum, and bladder or even within the thoracic cavity (10). Approximately 10%–15% of all women of reproductive age have the condition, but it is most common in women with infertility (25%–50%) and chronic pelvic pain (70%–85%) (5*,*^9^). The most common symptoms include chronic pelvic pain, acute pelvic pain, and infertility, but women with endometriosis are susceptible to a wide variety of other comorbidities, including cancer, lupus, rheumatoid arthritis, and cardiovascular disease (11). The societal and economic costs of endometriosis are alarming: those afflicted experience stigma, diminished quality of life, and reduced work productivity, and its management is responsible for about $70 billion in yearly health expenditures in the United States alone (3*,*^11^).

The current standard of care for the diagnosis of endometriosis is exploratory laparoscopy (12). This approach is invasive, painful, expensive, and prone to sampling and operator biases, factors that have combined to create long diagnostic delays for the condition (5*,*^9^). Anatomic imaging also plays an important role in the care of patients with the condition. Ultrasonography is frequently used as a prediagnostic tool to visualize lesions before surgical diagnosis and resection, but it can exhibit suboptimal sensitivity and accuracy in the context of smaller lesions (13). MRI has also been used to determine the size, location, and infiltrative stage of large lesions before surgical resection, especially in cases of deep infiltrating endometriosis (14). Unfortunately, MRI has proven suboptimal for the detection of small superficial lesions (i.e., <5 mm beneath the surface of the peritoneum) (14).

In light of the clear limitations of extant methods for the detection of endometriosis, researchers have begun to explore the possibility of using nuclear imaging—and PET in particular—for the visualization of endometriosis. Silveira et al., for example, used a rat model of the disease to determine the uptake of [^18^F]fluorocholine in superficial lesions. Although the activity concentration of the tracer in implanted endometriosis tissue was found to be 3-fold higher than that in the muscle and peritoneum, the biodistribution data revealed very high uptake in several healthy tissues, most notably (and problematically) the ovaries (15). More recently, Amartuvshin et al. probed the value of a ^64^Cu-labeled variant of the vascular endothelial growth factor–targeting antibody bevacizumab for the delineation of subcutaneous endometriosis lesions. Unfortunately, in vivo experiments revealed low activity concentrations in the target tissue and high levels of uptake in healthy tissues (16).

**FIGURE 2.**
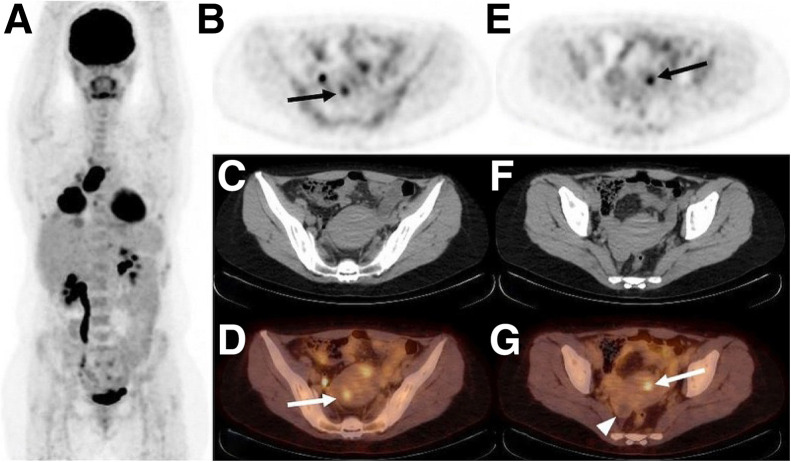
[^18^F]FDG PET/CT of 47-y-old woman with sarcomatous lung cancer and 11-y history of endometriosis at time of imaging. (A) Intense [^18^F]FDG uptake can be seen in primary lung cancer lesions as well as lymph node and pleural metastases. (B–G) Two foci of increased [^18^F]FDG avidity can be seen in endometriosis lesions in uterine wall via PET (B and E) and PET/CT (D and G) (arrows) but are not clearly visualized by CT alone (C and F). No [^18^F]FDG uptake was observed in known right ovarian endometrioma (arrowhead). (Reprinted with permission of (17).)

Shifting to the clinic, a handful of trials have focused on the potential of a trio of commonly used PET tracers—[^18^F]FDG, 16α-[^18^F]fluoro-17β-estradiol ([^18^F]FES), and [^68^Ga]Ga-DOTATATE—for the detection of endometriosis. Balogova et al., for example, performed [^18^F]FDG PET/CT on 18 patients with known or suspected endometriosis and found that the radiotracer effectively delineated only 11 of 20 (55%) confirmed lesions (Fig. 2) (17). More recently, a team at the Université Libre de Bruxelles in Belgium performed [^68^Ga]Ga-DOTATATE PET/CT on a cohort of patients (*n* = 12) scheduled for exploratory laparoscopy for suspected endometriosis. Although the authors ultimately concluded that the radiotracer was effective for the visualization of deep infiltrating endometriosis (i.e., the sensitivity and specificity were 57% and 80%, respectively), no uptake was observed in superficial peritoneal endometriosis or ovarian endometriomas (18). Without question, the most promising clinical results have been obtained with [^18^F]FES. In 2016, Cosma et al. performed [^18^F]FES PET on a group of patients (*n* = 4) with suspected endometriosis who also underwent MRI and laparoscopic excision coupled with histology. The data demonstrated that [^18^F]FES PET/CT fully agreed with histology and showed greater accuracy than MRI for lesions: PET/CT correctly identified 9 of 9 lesions, whereas MRI produced 3 false negatives and 3 false positives (3).

Molecular imaging could also play a key role in surgeries for endometriosis. Indeed, intraoperative imaging tools capable of visualizing endometriosis lesions could reduce operator bias during both laparoscopic diagnoses and surgical resections (19). Along these lines, a 2021 study by Al-Taher et al. compared intraoperative near-infrared fluorescence imaging with indocyanine green and conventional white-light laparoscopy for the delineation of endometriosis lesions in a cohort of 15 patients (20). The investigators determined that the positive predictive values for near-infrared fluorescence and white-light imaging were 69% and 64%, respectively, suggesting that the former could have modest value in the context of surgical resections. Interestingly, 2 other recent studies have sought to use intraoperative imaging to illuminate healthy tissues and thus differentiate endometriosis lesions. Aleksandrov et al. used indocyanine green to identify healthy rectum tissue when removing deep infiltrating endometriosis from the bowels (21), whereas Thigpen et al. used indocyanine green to differentiate between the ureters and endometriosis lesions during surgery (22). In both cases, the authors concluded that intraoperative near-infrared fluorescence imaging aided in the safe resection of the diseased tissue while reducing complications; however, the approximate location of the lesions still needs to be known before surgery.

Taken together, the PET and near-infrared fluorescence studies described in this section clearly suggest that molecular imaging could play important roles in the diagnosis and treatment of endometriosis. However, it is impossible to deny that the results have been middling at best, a problem that we suspect is related to the reuse of established imaging agents (i.e., [^18^F]FDG, [^18^F]fluorocholine, and indocyanine green). It is likely that novel probes with specificity for endometrial tissue will offer the best chance for the development of clinically effective tools.

## ADENOMYOSIS

Adenomyosis is a chronic disease similar to endometriosis that is characterized by the invasion and growth of endometrial tissue within the myometrium, the muscular middle layer of the uterus. Adenomyosis, although asymptomatic in almost a third of patients, can cause pelvic pain, infertility, and heavy menstrual bleeding (23). Ultrasonography and MRI are the primary diagnostic tools for the condition, but these modalities exhibit suboptimal accuracy, especially when adenomyosis is not suspected (23). For example, pelvic ultrasound has a sensitivity of 10.9% and a specificity of 98.3%. MRI fares slightly better, with a sensitivity of 29.7% and a specificity of 85.3%, numbers that improve slightly (though not overwhelmingly) during direct evaluations for the condition (24). New approaches to the diagnostic imaging of adenomyosis are thus needed, but there have been only a handful of reports on the use of nuclear imaging for the condition. And indeed, most of these have described the incidental uptake of radiopharmaceuticals in lesions. For example, a pair of reports that focused on cervical cancer and carcinomatosis has found that adenomyosis lesions can be [^18^F]FDG-avid, complicating the identification of intrauterine metastases via [^18^F]FDG PET (25*,*^26^). Furthermore, the aforementioned study that focused on the delineation of endometriosis with [^68^Ga]Ga-DOTATATE also found that adenomyosis lesions exhibited increased accretion of the radiolabeled peptide as well (18). Finally, a single case report by Wu et al. from 2014 described the visualization of pyoadenomyosis—a rare complication of adenomyosis—via SPECT with [^67^Ga]Ga^3+^ (27). In sum, the data clearly underscore that the stage is set for new work in this area, particularly the development of dedicated probes specific for adenomyosis.

## UTERINE FIBROIDS

Uterine fibroids, or leiomyomas, are benign clonal neoplasms of the uterus that occur in about 70% of women and disproportionately affect Black women (28). Though typically asymptomatic, these smooth muscle tumors can cause a wide range of symptoms—including pelvic pain, abnormal menstrual bleeding, and urinary and gastrointestinal issues—and can be associated with infertility and other adverse pregnancy outcomes (4*,*^29^). A variety of approaches has been used for the treatment of uterine fibroids, including myomectomy, radiofrequency ablation, uterine fibroid embolization, and hormonal birth control. Hysterectomy also remains a common treatment for the condition, with fibroids accounting for over one third of such procedures every year (4). Uterine fibroids are typically identified via ultrasonography, with postoperative histology providing a definitive diagnosis. Transvaginal sonography is inexpensive and exhibits high sensitivity (99%) and specificity (91%) that can be improved on even further with the addition of saline infusion sonohysterography for fibroids touching the uterine lining (30). MRI can also be used for diagnosis and offers sensitivity and specificity close to 100%, though it is significantly more expensive. That said, MRI can be useful in patients who are unsuitable for transvaginal sonography or saline infusion sonohysterography or who need preoperative mapping for myomectomy (7).

**FIGURE 3.**
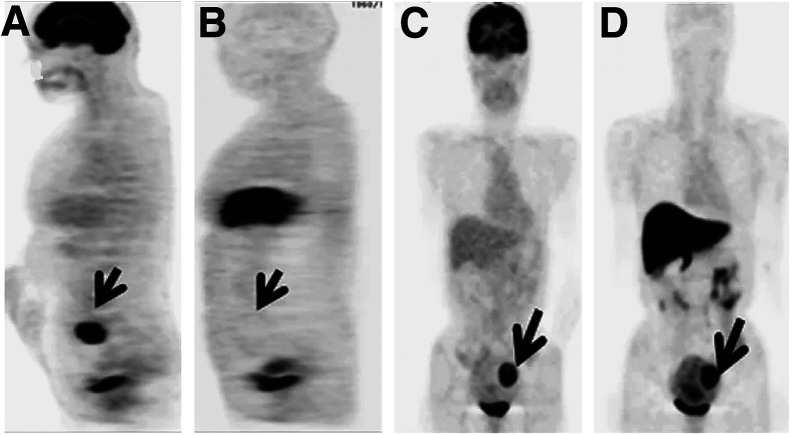
[^3^F]FDG and [^18^F]FES PET of 75-y-old woman with leiomyosarcoma (A and B, arrows) and 52-y-old woman with uncomplicated benign leiomyoma (C and D, arrows). In former, primary tumor in uterus accumulated [^18^F]FDG (A) but was negative for [^18^F]FES (B), and postoperative histopathologic results confirmed leiomyosarcoma. In latter, lesions were avid for both [^18^F]FDG (C) and [^18^F]FES (D), and postoperative histopathologic results confirmed uncomplicated leiomyoma and adenomyosis. (Reprinted with permission of (35).)

Existing methods for the detection of uterine fibroids are clearly sufficient. However, clinical studies on the visualization of leiomyomas with extant and emerging PET tracers are critical, especially in the context of differentiating between fibroids and malignant lesions. For example, a handful of studies has combined to show that leiomyomas of younger and premenopausal women can be especially [^18^F]FDG-avid, creating the possibility that these fibroids could be misidentified as leiomyosarcomas or endometrial carcinomas (31*–*33). A pair of clinical trials from Japan suggests that [^18^F]FES and [^18^F]FDG could play complementary roles in the differentiation of leiomyomas from endometrial carcinomas, leiomyosarcomas, and endometrial hyperplasia (34*,*^35^). More specifically, a malignant leiomyosarcoma showed positive [^18^F]FDG accumulation (SUV, 10.5) and negative [^18^F]FES accumulation (SUV, 1.0) in PET images, whereas a benign leiomyoma showed similarly positive uptake for both PET tracers (Fig. 3) (35). Finally, incidental uptake in uterine fibroids has been observed with several other PET tracers—i.e., [^68^Ga]Ga-DOTATATE, Al[^18^F]F-NOTA-FAPI, and [^18^F]NaF—though more comprehensive follow-up studies have yet to be performed (36*–*38). In the end, it is unlikely that molecular imaging will play a key standalone role in the detection of uterine fibroids given the efficacy of existing diagnostic methods. That said, imaging—and PET in particular—could be a valuable tool for differentiating between benign fibroids and more dangerous leiomyosarcomas. More broadly, as the use of a library of radiopharmaceuticals applied in the clinic grows, it will be important to understand the uptake of common radiopharmaceuticals in leiomyomas to prevent false-positive cancer diagnoses.

## PCOS

PCOS is an endocrine disorder that affects roughly 5%–15% of reproductive-aged women and can cause excess body hair, acne, weight gain, and infertility. Although the condition can be managed via hormone treatments and weight-loss drugs (as diabetes and insulin resistance are common comorbidities), accurate diagnoses are critical since many of these symptoms can be associated with other etiologies (4). Along these lines, PCOS is diagnosed when a patient satisfies 2 of the following 3 criteria: oligomenorrhea or anovulation, often leading to irregular periods; clinical or biochemical signs of hyperandrogenism; and polycystic ovaries on ultrasound. In light of this largely effective diagnostic paradigm, molecular imaging likely has a greater future role to play in understanding the condition rather than delineating it. Several studies have sought to use [^18^F]FDG to image brown adipose tissue levels in women with PCOS, as brown adipose tissue is a highly energetic tissue that is often functionally abnormal in patients with PCOS. In a 2019 trial, for example, [^18^F]FDG PET revealed that women with PCOS exhibited lower brown adipose tissue levels than those without the condition (39). A year later, similar results were obtained in a [^18^F]FDG PET study that compared a cohort of women receiving metformin for PCOS and those without the condition, cementing low levels of brown adipose tissue as a common characteristic of PCOS and opening the door for [^18^F]FDG as a tool for monitoring the treatment of the condition (40). Interestingly, this was not the first attempt at using nuclear imaging in the service of treating PCOS. In 2010, Priyadarshani et al. determined the biodistribution of a ^99m^Tc-labeled variant of noscapine in a rat model of PCOS, as the alkaloid was (at the time) being explored as a possible therapeutic for the condition (41). The results were reasonably promising—the ^99m^Tc-labeled noscapine produced 0.9 %ID/g in ovarian cysts compared with 0.06 %ID/g for a nonspecific control compound—but no follow-up studies have been published. In the future, it is unlikely that a nuclear imaging agent will be needed as a diagnostic tool for PCOS, but it is almost certain that both established tracers (e.g., [^18^F]FDG) and novel cyst-specific probes could play important roles in improving our understanding of the disease.

## ADNEXAL MASSES AND OVARIAN CYSTS

Adnexal masses are growths found around the ovaries, uterus, or fallopian tubes, including functional ovarian cysts, endometriomas, and teratomas. The accurate detection and identification of these lesions are critical, as they can interfere with cancer diagnoses and, in rare cases, become malignant themselves (4). Although most are asymptomatic, adnexal masses can cause pelvic pain on cyst enlargement, cyst rupture, or ovarian torsion (4). For patients who do experience symptomatic pain, diagnosis is typically performed via ultrasonography—most efficiently when performed by a sonographer using subjective pattern recognition—but other imaging modalities (e.g., pelvic MRI) may be leveraged for complementary information.

**FIGURE 4.**
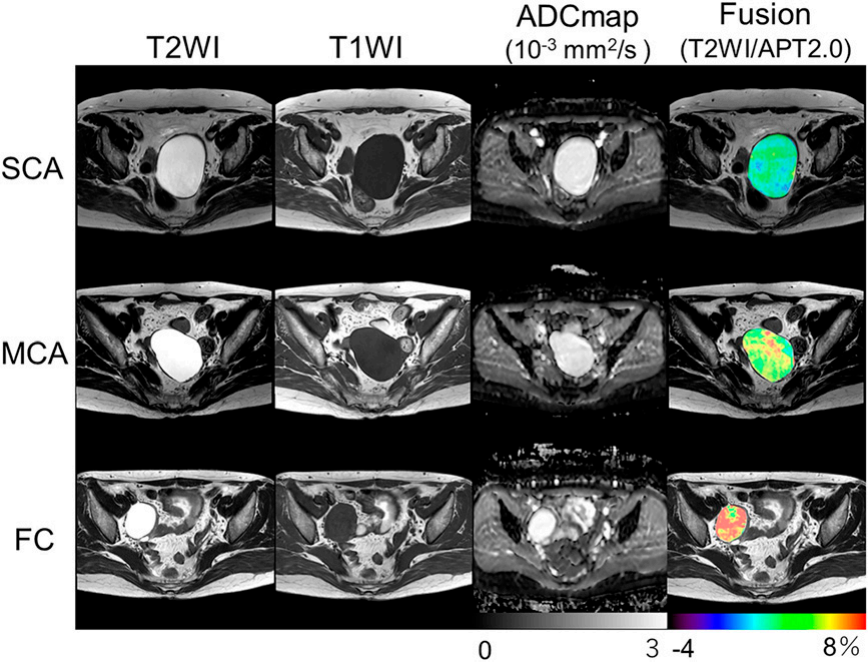
Typical T2-weighted images (T2WI), T1-weighted images (T1WI), apparent diffusion coefficient (ADC) maps, and amide proton transfer maps (APT) (overlaid on T2WI) of serous cystadenoma (SCA) (top), mucinous cystadenoma (MCA) (middle), and functional cyst (FC) (bottom). All 3 cystic lesions showed similar signal intensities in T1WI and T2WI as well as similar ADC values. However, amide proton transfer values were clearly different among trio of cysts: low in serous cystadenoma (2.25%), moderate in mucinous cystadenoma (5.03%), and very high in functional cyst (7.38%). Color bar indicates amide proton transfer signal (%). (Reprinted with permission of (44).)

Functional ovarian cysts are a class of adnexal masses that can develop on the ovaries, including follicular, corpus luteum, and theca lutein cysts. The most common approach to diagnosing these cysts is via transvaginal ultrasonography, an approach that offers a sensitivity of 93.5% and a specificity of 91.5% (42). Although these values are high, clinicians have nonetheless looked toward molecular imaging for complementary diagnostic tools. In a 2021 metaanalysis of 27 reports on the differentiation between benign and malignant adnexal masses via [^18^F]FDG PET and MRI, the average sensitivity and specificity were 94% and 86%, respectively, for [^18^F]FDG PET and 92% and 85%, respectively, for MRI (43). In a trial performed in Japan, an emergent type of MRI—amide proton transfer MRI—proved quite useful for the detection and classification of a variety of ovarian cysts. Indeed, amide proton transfer MRI provided correct diagnoses of each type of ovarian cyst, including serous cystadenomas, mucinous cystadenomas, and functional cysts, without additional follow-up studies (Fig. 4) (44).

MRI has also been found effective for identifying other benign adnexal masses, such as teratomas. In a 2015 study of 26 patients, for example, MRI proved superior to [^18^F]FDG PET for distinguishing between benign (mature) and malignant (immature) teratomas (45). A previous study with [^67^Ga]Ga SPECT provided similar results (46). The dense and fibrous nature of Brenner tumors—an ovarian lesion that can be benign or malignant—facilitates ready visualization via MRI, making it the current diagnostic gold standard (47). There may be room for nuclear imaging, however. To wit, one recent study suggests that malignant Brenner tumors may exhibit higher uptake of [^18^F]FDG than their benign cousins, aiding in the differentiation between the two (48).

## CONCLUSION

In assembling this review, we were taken aback (though not entirely surprised) by the degree to which benign gynecological pathologies remain understudied despite their global prevalence (1*,*^5^). We are heartened, however, to see that molecular imaging research in this area has increased over the last decade, as we believe that molecular imaging has the potential to improve patient outcomes. In conditions for which current diagnostic paradigms are inadequate—such as endometriosis and adenomyosis—molecular imaging could become a pivotal diagnostic tool. Yet molecular imaging could still have a part to play in cases for which standard-of-care diagnostic technologies are sufficient. In PCOS, for example, molecular imaging could help us better understand the syndrome’s diverse constellation of symptoms, whereas in the context of benign adnexal masses, it could be deployed to better differentiate between benign and malignant lesions. Going forward, we are eager to see what the next decade has in store for research at the intersection of these 2 areas. Although there are a plethora of viable paths for work, we believe that the development of novel imaging probes that specifically target these conditions will be especially critical. In the end, details aside, we are simply hopeful that advances in our field can help the millions of women with these conditions.

## DISCLOSURE

No potential conflict of interest relevant to this article was reported.
